# Role of perceived heart risk factors by outpatient population in predicting cardiovascular risk

**DOI:** 10.15171/jcvtr.2019.18

**Published:** 2019-05-23

**Authors:** Ali Soroush, Nasim Shams-Alizadeh, Afsoon Vahdat, Zeinab Mohebi, Mozhgan Saeidi, Saeid Komasi

**Affiliations:** ^1^Heart Research Center, Imam Ali Hospital, Kermanshah University of Medical Sciences. Kermanshah, Iran; ^2^Lifestyle Modification Research Center, Imam Reza Hospital, Kermanshah University of Medical Sciences, Kermanshah, Iran; ^3^Clinical Research Development Center, Imam Reza Hospital, Kermanshah University of Medical Sciences, Kermanshah, Iran; ^4^Cardiac Rehabilitation Center, Imam Ali Hospital, Kermanshah University of Medical Sciences, Kermanshah, Iran

**Keywords:** Cardiovascular Disease, Heart Risk, Lifestyle, Perception, Risk Factors

## Abstract

***Introduction:*** Regarding the expanding population in developing countries who are at risk for cardiovascular diseases (CVDs), identification and management of effective factors are important in reducing the risk of CVDs. So, the present study aimed to assess the role of perceived heart risk factors (PHRFs) in the prediction of cardiovascular risk among outpatient patients.

***Methods:*** The samples of this cross-sectional study included 150 outpatient patients who attend the clinic of Imam Reza hospital during October-December 2016. The participants were completed the Perceived Heart Risk Factors Scale (PHRFS) and Cardiovascular Risk Assessment Questionnaire (CRAQ). Data analyzed through Pearson correlation and multiple regression analyses.

***Results:*** Based on the findings, 28%, 40%, 22.7%, and 9.3% of patients were low, medium, high, and severely high-risk, respectively. The strongest predictors of the cardiovascular risk were physiological (β=-0.273; *P*=0.004), psychological (β=0.236; *P*=0.020), and biological risk factors (β=0.209; *P*=0.016), respectively. In addition, the strongest predictor of the lifestyle risk was physiological risk factors (β=-0.264; *P*=0.007). Other variables do not play a significant role in predict the lifestyle risk (*P*>0.05). Our model was able to explain 9.2% of cardiovascular risk variance and 5.7% of cardiovascular risk caused by lifestyle variance.

***Conclusion:*** The higher patients’ perception about biological and psychological risk factors is concerned as an alarm for increased cardiovascular risk while higher perception about physiological risk factors is associated with reduced cardiovascular risk caused by lifestyle and total cardiovascular risk. The programs reducing cardiovascular risk should target the high-risk groups to save cost and time.

## Introduction

Cardiovascular diseases (CVDs) are most prevalent diseases in developing countries which led to mortality.^1^ In Iranian population, the prevalence of CVDs is higher than Western countries,^2^ and it is predicted that disability-adjusted life years related to CVDs will increase more than two-fold by 2025.^3^ The results of a recent cohort study in Iran showed that 186 to 584 cases of 100 000 individuals are predisposing for these diseases.^4^ On the other hand, more than 70% of the risk of CVDs and mortality caused by it is attributable to modifiable risk factors.^5^ It seems that this worrying situation concluded from failure in control of modifiable risk factors such as low physical activity, overweight, inappropriate nutrition, smoking, and unhealthy lifestyle.^6^

Based on the self-regulation model, perception about risk factors of disease and related knowledge of its etiology can impact on health behaviors.^7^ According to the recent reports, perception about cardiovascular risk factors included biological, environmental, physiological, behavioral, and psychological factors^7,8^ can predict future health behaviors.^9-11^ Correct perception of risk factors and individuals belief about the possibility of confront to health threat may be effective in following healthy lifestyle.^12^ Although, the results of a report indicating that at least 25% of individuals have not correct perception about cardiovascular risk factors.^13^ So, according to the great portion of at-risk people, their poor perception about risk factors and importance of initial prevention, the present study aimed to assess the role of perceived heart risk factors (PHRFs) in the prediction of cardiovascular risk among outpatient patients.

## Materials and Methods

### Design and context

**Figure 1:**
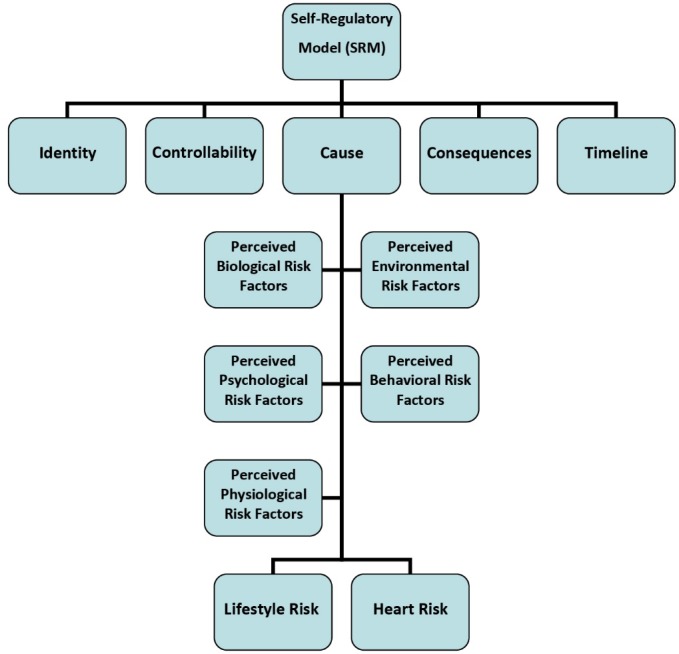
The conceptual model of the present study.

In this cross-sectional study, all patients who attend the outpatient clinic of Imam Reza hospital (Kermanshah, Iran) during October-December 2016 asked to participate. Imam Reza hospital is a general governmental hospital which has 750 beds and more than 10 inpatient wards. At average, 1500 patients are admitted and 15000 outpatients receive the diagnostic and treatment services monthly. Given that our study is based on the self-regulatory model, Figure 1 shows the conceptual model of the present study.

### Inclusion and exclusion criteria

Inclusion criteria included 18-85 years aged, having at least elementary education level, lack of cardiac surgery history or each kind of non-medical intervention, and no history of participation in a cardiac rehabilitation program. In addition, incomplete answer sheets were dropped after controlling and checking the responses.

### Participants

The participants included outpatients who attend the outpatient clinic of Imam Reza hospital during October-December 2016. At the time of data collection, approximately 400 people visited the outpatient clinic. At first, 200 of these were non-randomly selected; but 168 people remained after considering the inclusion criteria. In addition, 16 people did not want to participate in the study. So, the sample size included 152 patients. Also, the dropped questionnaires were two cases. Finally, 150 people stayed and enter to the final analysis. According to the formula ‘N>50+8m’, the minimum sample size of the present study concerned as 90 cases.^14^ Nevertheless, due to the lack of cooperation by some participants and attrition, 150 people were examined.

### Collecting data

The patients who had inclusion criteria were identified by the research team and they wrote written consent form and ensured about their secreted information then they fulfilled demographic data, medical history form, the Perceived Heart Risk Factors Scale (PHRFS), and Cardiovascular Risk Assessment Questionnaire (CRAQ). In the first step, a trained expert psychologist interviewed each patient and recorded their demographic information and medical histories in the research forms. Then, the PHRFS and CRAQ were distributed to the participants. The patients were asked to answer the questionnaires in the presence of the same psychologist interviewer. In addition, the interviewer tried to answer participants’ questions without influence on their answers. In the final step, the responses were collected and checked, and their data were recorded in the statistical software. As mentioned above, the patients wrote written consent form and ensured about their secreted information.

### Instruments

*Demographics and medical histories checklist:* This checklist included age, gender, marital status, education level, occupation, family history of CVDs and risk factors including current smoking, passive smoking, substance abuse, hypertension, diabetes, hyperlipidemia, and MI history. All data were registered in the checklist by a researcher.

*The Perceived Heart Risk Factors Scale (PHRFS):* This questionnaire designed and made by Saeidi and Komasi.^8^ It has 5 subscales included biological factors (items of 1-3 related to genetics, aging, male/female gender), environmental factors (items of 4-8 related to smoke and toxic substances, polluted water and air, dust, war between countries, passive smoking), behavioral factors (items of 9-14 related to smoking, drug abuse, drinking, malnutrition, physical inactivity, physical work pressure), psychological factors (items of 15-21 related to psychological stress, anger and rage, emotions, sadness and grief, depression, marital discord, discomfort due to financial problems), and physiological risk factors (items of 22-25 related to high cholesterol, hypertension, diabetes, obesity). Each of items scored in a Likert system (never: 0, a little: 1, somewhat: 2, great: 3, and very great: 4) so, the maximum scores for each subscale are 12, 20, 24, 28, and 16 respectively and the total score is 0-100. Internal consistency of items of the whole scale is 0.933. The content validity approved by Lawshe method. In addition, the result of factor analysis showed that it has appropriate validity.^8^

*Cardiovascular Risk Assessment Questionnaire (CRAQ):* This questionnaire, originally developed by the *Australia and New Zealand Health World*,^15^ has 2 parts which the first part is fulfilled by a patient while the second part is fulfilled by a physician. The first part has 10 subscales included risk related to age (score range from 0 to 140), cardiovascular history(score range from 0 to 250), CVD in family (score range from 0 to 45), healthy/unhealthy lifestyle (e.g. physical activity, smoking, passive smoking, alcohol abuse, and environment; score range from -35 to 150), stress and its management (score range from -19 to 330), sleep duration and its disorders (score range from 0 to 29), bowel toxicity (e.g. regularly experience lower abdominal pain, gas, bloating, diarrhea, constipation, straining when passing bowel motions, excessively smelly stools and/or a feeling that your bowels do not completely empty, and taken the oral contraceptive pill and antibiotics in the last year; score range from 0 to 30), blood sugar and diabetes (score range from 0 to 110), infection and pain (score range from 0 to 60), and healthy/unhealthy nutrition (score range from -23 to 48). There is higher score mean more cardiovascular risk. Negative scores indicate a decreasing effect on cardiovascular risk (e.g. healthy lifestyle or nutrition). The second part included nine components related to risk of lipids (e.g. triglycerides, HDL, LDL, lipoprotein; score range from -15 to 155), blood pressure (score range from 0 to 60), infection and pain (e.g. C-reactive protein, homocysteine, fibrinogen, urinary pH; score range from 0 to 239), bowel and liver toxicity (score range from 0 to 10), stress (e.g. abnormal cortisol levels; score range from 0 to 12), thyroid function (score range from 0 to 20), blood sugar (score range from 0 to 50), waist measurement (score range from 0 to 50), and weight management (score range from 0 to 25). The cardiovascular risk indicated as low risk (-88 to 100), medium risk (101 to 220), high-risk (221 to 350), and severely high-risk (351 and more).^15^ Although, we used only the first part of this questionnaire. In the present study, Cronbach’s alpha for total scale (internal consistency) was 0.811.

### Statistical analysis

Demographic data and medical histories of patients included gender, education, occupation, marital status, smoking, substance and alcohol abuse, myocardial infarction, hypertension, diabetes, and hyperlipidemia reported based on percentage. Also, the means and standard deviations of continuous data included age, the PHRFS, and CRAQ were reported. In the main analysis, after approving of the lack of rollout of the needed pre-assumptions,^14^ a multiple regression was conducted. In the first model, the regression analysis used to assess the role of PHRFs in the prediction of the level of cardiovascular risk. In the second model, this method was used to assess the role of PHRFs in the prediction of the level of cardiovascular risk induced by lifestyle. The analysis conducted by Statistical Package for Social Science (SPSS) version 20. All statistical tests were 2-sided; a *P* value ≤ 0.05 was considered significant.

## Results

Based on the findings, 28%, 40%, 22.7%, and 9.3% of patients were low risk, medium risk, high-risk, and severely high-risk respectively. Table 1 indicates demographic data and risk factors.

**Table 1:** Demographics and risk factors of the samples

**Variables**	**Total (n = 150)**
Sex (%) Male Female	98 (65.3) 52 (34.7)
Marital status (%)	
Single	43 (28.7)
Marriage	92 (61.3)
Divorced	15 (10.0)
Education (%)	
Under diploma	49 (32.6)
Diploma	55 (36.7)
Academic	46 (30.7)
Job (%)	
Employee	30 (20.0)
Self-employee	29 (19.3)
Housekeeper	49 (32.7)
Retired	16 (10.7)
Unemployed	25 (17.3)
Smoking (%)	
Never	134 (89.3)
Cessation	5 (3.3)
Active	11 (7.4)
Substance abuse (%)	
Never	144 (96.0)
Cessation	3 (2.0)
Active	3 (2.0)
Drinking (%)	
Never	139 (92.7)
Cessation	6 (4.0)
Active	5 (3.3)
Risk factors (%)	
Hypertension	18 (12.0)
Diabetes	8 (5.3)
Hyperlipidemia	17 (11.3)
Myocardial Infarction history	5 (3.3)
Age, year (M ± SD)	37.9 ± 13.8

The data are represented as frequency (percentage) or mean (standard deviation).

The mean and standard deviation of scores of CRAQ and PHRFS can be seen in Table 2. As can be seen, the total score of CRAQ is 183.35±121.06. Based on the cardiovascular risk classification mentioned in the instruments section, this score is placed on the medium risk (that’s mean scores 101-220). Also, in the table, the mean scores of CRAQ subscales are comparable to the scores range. Table 2 shows that the participants are high-risk solely in the psychological stress factor. In addition, risk related to cardiovascular history, unhealthy lifestyle, and bowel toxicity risk is moderate.

**Table 2:** The scores of cardiovascular risk assessed by CRAQ and PHRFS subscales

**Variable**	**Score (M ± SD)**	**Cardiovascular risk category**
**CRAQ score**		
Age risk (range of 0 to 140)	26.96 ± 39.77	Not a modifiable risk factor
Cardiovascular history risk (0 to 250)	37.67 ± 68.77	Low: (0 to 30) Medium: (31 to 50) High: (51 and above)
Family history risk (0 to 40)	9.50 ± 12.34	Not a modifiable risk factor
Lifestyle risk (-35 to 150)	18.70 ± 28.38	Low: (-35 to -10) Medium: (-9 to 21) High: (22 and above)
Stress risk (-19 to 330)	55.06 ± 36.45	Low: (-19 to 20) Medium: (21 to 40) High: (41 and above)
Sleep risk (0 to 29)	4.76 ± 3.94	Low: (0 to 5) Medium: (6 to 11) High: (12 and above)
Bowel toxicity risk (0 to 30)	5.06 ± 5.13	Low: (0 to 3) Medium: (4 to 9) High: (10 and above)
Blood glucose risk (0 to 110)	10.33 ± 22.06	Low: (0 to 19) Medium: (20 to 49) High: (50 and above)
Infection and pain risk (0 to 60)	10.90 ± 12.42	Low: (0 to 19) Medium: (20 to 42) High: (43 and above)
Nutrition risk (-23 to 48)	4.41 ± 6.10	Low: (-19 to 6) Medium: (7 to 13) High: (14 and above)
Total cardiovascular risk	183.35 ± 121.06	Low: (-88 to 100) Medium: (101 to 220) High: (221to 350) Very high: (351 and above)
**PHRFS Score**		
Biological risk factors (range of 0 to 12)	6.38 ± 2.32	
Environmental risk factors (0 to 20)	14.19 ± 3.71	
Behavioral risk factors (0 to 24)	16.89 ± 3.82	
Psychological risk factors (0 to 28)	19.84 ± 4.73	
Physiological risk factors (0 to 16)	12.15 ± 2.75	

Abbreviation: CRAQ,cardiovascular Risk Assessment Questionnaire; PHRFS, Perceived Heart Risk Factors Scale.

Table 3 shows the Pearson correlations between components of the CRAQ and PHRFS. Table 4 indicates the Pearson correlation coefficients between variables and the results of multiple regression analysis to predict cardiovascular risk among cases. There is an inverse relationship only between physiological PHRFs with totally cardiovascular risk (r= -0.136, *P *= 0.048), as well as heart risk induced by lifestyle risk (r= -0.167, *P *= 0.020). No significant relationship was found between the other variables (*P*>0.05). The results of the table also indicate that biological and psychological PHRFs can predict increased cardiovascular risk directly, while physiological PHRFs can predict it indirectly. The strongest predictors in the cardiovascular risk model were physiological (β= -0.273; *P *= 0.004), psychological (β* *= 0.236; *P *= 0.020), and biological PHRFs (β* *= 0.209; *P *= 0.016), respectively. Generally, the PHRFs model was able to explain 9.2% of totally cardiovascular risk variance (R^2^=0.092; F=2.916; *P *= 0.015).

**Table 3:** The Pearson correlations between subscales of the CRAQ and PHRFS

**Variable**	**1**	**2**	**3**	**4**	**5**	**6**	**7**	**8**	**9**	**10**	**11**	**12**	**13**	**14**	**15**
**CRAQ subscales**															
1. Age	-														
2. Cardiovascular history	0.106	-													
3. Family history	0.085	0.127	-												
4. Lifestyle	0.050	0.044	0.088	-											
5. Stress	0.008	**0.163**	0.118	0.085	-										
6. Sleep	**0.369**	0.160	0.105	**0.285**	**0.262**	-									
7. Bowel toxicity	0.154	-0.016	**0.166**	**0.194**	**0.212**	**0.280**	-								
8. Blood glucose	**0.239**	**0.169**	**0.207**	0.023	-0.016	**0.217**	**0.190**	-							
9. Infection and pain	**0.241**	**0.204**	**0.197**	0.069	**0.264**	**0.325**	**0.390**	0.154	-						
10. Nutrition	0.124	0.014	0.093	**0.229**	-0.020	0.114	0.028	0.079	0.026	-					
11. Total risk	**0.505**	**0.732**	**0.331**	**0.351**	**0.469**	**0.491**	**0.295**	**0.413**	**0.469**	**0.178**	-				
**PHRFS subscales**															
12. Biological factors	-0.038	0.116	0.101	-0.004	0.023	0.058	0.035	0.155	0.118	**0.196**	0.123	-			
13. Environmental factors	-0.026	0.018	-0.006	0.014	-0.011	-0.002	0.038	-0.015	-0.001	-0.055	-0.003	**0.249**	-		
14. Behavioral risk factors	-0.068	0.083	0.004	0.079	0.009	-0.101	-0.062	-0.073	-0.085	-0.052	0.016	0.156	**0.708**	-	
15. Psychological factors	0.011	0.139	0.109	-0.011	-0.002	0.102	-0.036	0.083	0.089	0.031	0.118	0.090	**0.543**	**0.543**	-
16. Physiological factors	-0.065	-0.072	0.036	-0.152	-0.113	**-0.191**	-0.141	0.065	-0.091	0.032	-0.136	**0.319**	**0.397**	**0.439**	**0.385**

Abbreviation: CRAQ=cardiovascular Risk Assessment Questionnaire, PHRFS=Perceived Heart Risk Factors Scale. Boldface indicates statistically significant (*P* < 0.05).

**Table 4:** The Pearson correlations and regression model to predict cardiovascular risk and lifestyle risk assessed by CRAQ

**Predictors**	**Cardiovascular Risk**		**B**	**β** ^a^	**t**	***P*** ** value**
	**r**	***P***				
**PHRFS subscales (Model 1)**						
Biological risk factors	0.123	0.067	10.86	0.209	2.442	0.016
Environmental risk factors	- 0.003	0.487	- 3.69	- 0.113	- 0.949	0.344
Behavioral risk factors	0.016	0.424	1.73	0.055	0.460	0.646
Psychological risk factors	0.118	0.075	6.03	0.236	2.360	0.020
Physiological risk factors	- 0.136	0.048	- 12.03	- 0.273	- 2.903	0.004
**PHRFS subscales (Model 2)**	**Lifestyle Risk** ^b^					
Biological risk factors	0.039	0.318	2.34	0.111	1.271	0.206
Environmental risk factors	- 0.007	0.467	- 0.96	- 0.073	- 0.599	0.550
Behavioral risk factors	0.046	0.290	2.31	0.180	1.478	0.141
Psychological risk factors	- 0.004	0.479	0.30	0.029	0.288	0.774
Physiological risk factors	- 0.167	0.020	- 4.72	- 0.264	- 2.751	0.007

Abbreviations: CRAQ=cardiovascular Risk Assessment Questionnaire, PHRFS=Perceived Heart Risk Factors Scale. ^a^ The higher beta coefficients represent the stronger role of a variable in predicting the criterion variable. ^b^ Cardiovascular risk induced by lifestyle included psychological stress and nutrition. Summary of the model 1: R = 0.303, R2 = 0.092, F = 2.916, P = 0.015. Summary of the model 2: R = 0.239, R2 = 0.057, F = 1.741, P = 0.129.

Table 4 also indicates physiological risk factors only can predict the increased cardiovascular risk induced by lifestyle indirectly. In fact, the strongest predictor in the lifestyle risk model was physiological PHRFs (β= -0.264; *P *= 0.007). Other variables do not play a significant role in predicting the lifestyle risk (*P *> 0.05). Generally, the PHRFs model was able to explain 5.7% of totally lifestyle risk variance (R^2^ = 0.057; F* *= 1.741; *P *= 0.129).

## Discussion

### Main findings

- Seventy-two percent of the outpatients have a medium or higher cardiovascular risk.
- Psychological stress is the most important sub-factor of cardiovascular risk. The samples are medium risk in terms of cardiovascular history, unhealthy lifestyle, and bowel toxicity.
- Higher biological and psychological PHRFs can directly predict increased cardiovascular risk.
- Higher physiological PHRFs are associated with decreased totally cardiovascular risk and risk induced by lifestyle.

The current study showed that 72% of the samples have a medium or higher cardiovascular risk. This percentage of the population at risk of CVDs is much higher than the results of similar studies in the outpatient population.^16,17^ Although a wide range of the general population in developing countries is at risk for CVDs,^1^ approximately 40% of them minimizes the risk of these diseases.^18^ Our results indicated that 28%, 40%, 22.7%, and 9.3% of patients are low risk, medium risk, high-risk, and severely high-risk, respectively. It means that more than 1/3 of participants are medium risk and about 1/3 of samples are the severely high-risk for CVDs. It should not be noted that if the second part of the CRAQ was implemented, the cardiovascular risk probably increased in most participants. Generally, it seems that poor health literacy can impact on increased risk, poor self-controlling skills, and inappropriate health choices.^18^

Other finding shows that psychological stress, cardiovascular history, unhealthy lifestyle, and bowel toxicity are the most important sub-factors of cardiovascular risk. This finding is consistent with previous studies.^19,20-22^ More than 96% of coronary patients suffer from moderate to severe stress.^19^ in addition, prior cardiac disease, lifestyle components such as physical inactivity, smoking, alcohol drinking, abnormal BMI have an important role in increased risk of CVDs.^20,21^ Meanwhile, the results of a review study showed that bowel toxicity increases the risk of developing CVDs.^22^

Another finding showed that biological and psychological PHRFs can directly predict increased cardiovascular risk. In other words, higher perception about biological and psychological risk factors is associated with increased cardiovascular risk. In a lateral analysis of the present results indicated that patients with family history of CVDs have the higher perception about biological risk factors compared to cases without family history. So, it is obvious that higher perception about biological risk factors induced by real confronts with these risk factors. So, this situation leads to higher perception about these factors and increased cardiovascular risk. The results of the qualitative studies show that most at risk populations and even cardiovascular patients know the familial history as the main reason for their disease.^23^ They know themselves as at risk cases because of uncontrollable biological and hereditary nature of the disease.^24^

On the other hand, psychological and psychosocial risk factors such as emotional factors and chronic stressors are related severely to an unhealthy lifestyle and risk for development of CVDs.^25-27^ The results of recent review studies point to the role of psychological stress, anxiety, and depression, social isolation, and anger in the emergence of CVDs in the general population.^28-30^ These factors may increase 1.2 to 2.5 times the risk of CVDs. It seems that most of the people experience psychological factors and stresses caused by it. Despite people who are at risk for these psychological tensions and emotional problems have a higher perception about these risk factors, they are severely at risk for CVDs.^21-23^ In this line, the results of a reportindicate that there is a strong relation between PHRFs or causal attributions with actual risk factors.^27^ Furthermore, the psychological stress is the most important perceived risk factor in assessed populations.^1,31,32^ in addition, a new report in Iran shows that there is a significant relationship between psychological risk factors including stress assessed by PHRFS and heart risk perception.^33^

The present results showed that there is an inverse relationship between physiological PHRFs and totally cardiovascular risk and/or risk induced by lifestyle. In other words, higher perception about physiological risk factors is associated with decreased cardiovascular risk and risk induced by lifestyle. In a lateral analysis of the present results, we found that individuals who have the history of physiological risk factors received higher scores in physiological PHRFs. It means that histories related to hypertension, diabetes, hyperlipidemia, and obesity are associated with higher perception about physiological risk factors. Based on these considerations, it can be suggested that individuals who have an appropriate perception about the role of physiological risk factors (hypertension, diabetes, hyperlipidemia, and obesity) know themselves more at risk so they experience more stress and anxiety.^11^ Therefore, this feeling leads to healthy lifestyle and control and the decrease of cardiovascular risk.^6^

Finally, the current study indicates that there is no relationship between environmental and behavioral PHFRs with cardiovascular risk in the outpatients. Unlike previous studies that emphasized the importance of behavioral risk factors and behaviorally healthy lifestyles,^12,21^ our findings showed that higher perception about behavioral risk factors does not necessarily lead to a reduction in cardiovascular risk. In explaining this finding, it can be said that patients probably have the different level of awareness about CVDs risk factors. This perception and awareness may be the result of personal learning and experiences; because the results of a study in Iran showed that formal training has no effect on improving the perception of cardiac patients from behavioral risk factors.^34^ Also, a new report showed that unlike psychological and physiological risk factors, behavioral and environmental factors cannot predict heart risk perception.^33^

## Limitations

We only performed the first part of the CRAQ which includes a score range of -15 to 565. This caused the patient’s cardiovascular risk to be less than real size. In future studies, the implementation of the second part of the questionnaire can provide more accurate information about distribution of cardiovascular risk among outpatients population. Due to the small size of the sample and lack of follow-up, it is recommended that these cases be considered in future studies. In addition, the participants in the study were non-randomly selected from an outpatient clinic in western Iran. This challenge may lead to bias in the current results. Thus, in order to generalize these findings, future studies should be carried out in several centers in different parts of the country.

## Conclusion

The higher patients’ perception about biological and psychological risk factors is concerned as an alarm for increasing cardiovascular risk while higher perception about physiological risk factors is associated with reduced cardiovascular risk induced by lifestyle and total cardiovascular risk. The programs reducing cardiovascular risk should target the high-risk groups to save cost and time.

## Ethical approval

The patients wrote written consent form and ensured about their secreted information. This project also received an ethical code (ID: KUMS.REC.1396.227) by Kermanshah University of Medical Sciences.

## Competing interests

All authors declare no competing financial interests exist.

## Acknowledgment

We appreciate the Clinical Research Development Center of Imam Reza Hospital, Kermanshah University of Medical Sciences. This project was supported by Kermanshah University of Medical Sciences (ID: 96218).

## References

[1] Saeidi M, Soroush A, Komasi S, Moemeni K, Heydarpour B (2015). Attitudes toward cardiovascular disease risk factors among patients referred to a cardiac rehabilitation center: importance of psychological attitudes. Shiraz E-Med J.

[2] Ebrahimi M, Kazemi-Bajestani S, Ghayour-Mobarhan M, Ferns G (2011). Coronary artery disease and its risk factors status in Iran: a review. Iran Red Crescent Med J.

[3] Sadeghi M, Haghdoost AA, Bahrampour A, Dehghani M (2017). Modeling the Burden of Cardiovascular Diseases in Iran from 2005 to 2025: The Impact of Demographic Changes. Iran J Public Health.

[4] Talaei M, Sarrafzadegan N, Sadeghi M, Oveisgharan S, Marshall T, Thomas GN (2013). Incidence of cardiovascular diseases in an Iranian population: the Isfahan Cohort Study. Arch Iran Med.

[5] Sardarinia M, Akbarpour S, Lotfaliany M, Bagherzadeh-Khiabani F, Bozorgmanesh M, Sheikholeslami F (2016). Risk Factors for Incidence of Cardiovascular Diseases and All-Cause Mortality in a Middle Eastern Population over a Decade Follow-up: Tehran Lipid and Glucose Study. PLoS One.

[6] Chiuve SE, Cook NR, Shay CM, Rexrode KM, Albert CM, Manson JE (2014). Lifestyle‐based prediction model for the prevention of CVD: The healthy heart score. J Am Heart Assoc.

[7] Komasi S, Saeidi M (2016). Presentation of new classification of perceived risk factors and etiologies of cardiovascular diseases. ARYA Atheroscler.

[8] Saeidi M, Komasi S (2017). Reliability and validity of perceived heart risk factors scale. Ann Card Anaesth.

[9] Komasi S, Saeidi M (2015). Screening for depressive symptoms at the beginning of outpatient cardiac rehabilitation by assessed perceived risk factors by patients. Clin Med Rev Case Rep.

[10] Komasi S, Saeidi M (2016). A perceived risk factor may lead to increased anxiety and depression in cardiovascular patients. Jundishapur J Chronic Dis Care.

[11] Saeidi M, Komasi S, Heydarpour B, Momeni K, Zakiei A (2016). Those who perceive their disease as a physiological or psychological risk factor experience more anxiety at the beginning of the cardiac rehabilitation program. Res Cardiovasc Med.

[12] Abed MA, Khalil AA, Moser DK (2015). Awareness of modifiable acute myocardial infarction risk factors has little impact on risk perception for heart attack among vulnerable patients. Heart Lung.

[13] Panagiotakos DB, Georgousopoulou EN, Polychronopoulos E, Vassilakou T, Chrysohoou C, Pitsavos C (2013). Beliefs and attitudes regarding cardiovascular disease risk factors: a health survey in 10,141 Greek men and women (2006-2012). Int J Cardiol.

[14] Pallant J. SPSS survival manual: A step by step guide to data analysis using SPSS for Windows (Version 12). 2nd ed. Australia: Allen & Unwin. 2005. p 172-193.

[15] Australia and New Zealand Health World. Cardiovascular risk assessment questionnaire. Metagenics. 2013. https://www.metagenics.com.au and http://www.sydneynaturalhealth.com.au.

[16] Guo F, Hsieh E, Lv W, Han Y, Xie J, Li Y (2017). Cardiovascular disease risk among Chinese antiretroviral-naïve adults with advanced HIV disease. BMC Infect Dis.

[17] M Loukianov M, A Boytsov S, S Yakushin S, Yu Martsevich S, N Vorobyev A, V Zagrebelnyy A (2014). Outpatient registry of cardiovascular diseases (recvasa): Prospective follow-up data, estimation of risks and outcomes in patients with atrial fibrillation. Rational Pharmacother Cardiol.

[18] Webster R, Heeley E (2010). Perceptions of risk: understanding cardiovascular disease. Risk Manag Healthc Policy.

[19] Kurd BJ, Dar MI, Shoaib M, Malik L, Aijaz Z, Asif I (2014). Relationship between stress and coronary heart disease. Asian Cardiovasc Thorac Ann.

[20] Rockberg J, Jørgensen L, Taylor B, Sobocki P, Johansson G (2017). Risk of mortality and recurrent cardiovascular events in patients with acute coronary syndromes on high intensity statin treatment. Prev Med Rep.

[21] Udo T, Mun E-Y, Buckman JF, Vaschillo EG, Vaschillo B, Bates ME (2013). Potential side effects of unhealthy lifestyle choices and health risks on basal and reactive heart rate variability in college drinkers. J Studi Alcohol Drugs.

[22] Wu P, Jia F, Zhang B, Zhang P (2017). Risk of cardiovascular disease in inflammatory bowel disease. Exp Ther Med.

[23] Carroll C, Naylor E, Marsden P, Dornan T (2003). How do people with Type 2 diabetes perceive and respond to cardiovascular risk?. Diabetic Med.

[24] King KM, Norris CM, Knudtson ML, Ghali WA (2009). Risk-taking attitudes and their association with process and outcomes of cardiac care: a cohort study. BMC Cardiovasc Disord.

[25] Rozanski A, Blumenthal JA, Davidson KW, Saab PG, Kubzansky L (2005). The epidemiology, pathophysiology, and management of psychosocial risk factors in cardiac practice: The emerging field of behavioral cardiology. J Am Coll Cardiol.

[26] Menezes AR, Lavie CJ, Milani RV, O’Keefe J, Lavie TJ (2011). Psychological risk factors and cardiovascular disease: is it all in your head?. Postgrad Med.

[27] Glozier N, Tofler GH, Colquhoun DM, Bunker SJ, Clarke DM, Hare DL (05). Psychosocial risk factors for coronary heart disease. Med J Aust 2013.

[28] Cohen BE, Edmondson D, Kronish IM (2015). State of the art review: depression, stress, anxiety, and cardiovascular disease. Am J Hypertens.

[29] Steptoe A, Kivimäki M (2013). Stress and cardiovascular disease: an update on current knowledge. Annu Rev Public Health.

[30] Kivimäki M, Steptoe A (2017). Effects of stress on the development and progression of cardiovascular disease. Nature Rev Cardiol.

[31] Perkins-Porras L, Whitehead DL, Steptoe A (2006). Patients’ beliefs about the causes of heart disease: relationships with risk factors, sex and socio-economic status. Eur J Cardiovasc Prev Rehabil.

[32] Saeidi M, Komasi S, Soroush A, Zakiei A, Shakeri J (2014). Gender differences in patients’ beliefs about biological, environmental, behavioral, and psychological risk factors in a cardiac rehabilitation program. J Cardio-Thorac Med.

[33] Soroush A, Saeidi M, Komasi S (2018). Perceived Nonpsychological Etiologies of Cardiovascular Diseases are Unable to Predict Heart Risk Perception. Res Cardiovasc Med.

[34] Komasi S, Soroush A, Saeidi M, Brugnera A, Rabboni M, Fulcheri M (2018). Subjective correlates of stress management in outpatient cardiac rehabilitation: the predictive role of perceived heart risk factors. J Cardiovasc Thorac Res.

